# Retrospective, Observational Analysis on the Impact of SARS-CoV-2 Variant Omicron in Hospitalized Immunocompromised Patients in a German Hospital Network—The VISAGE Study

**DOI:** 10.3390/vaccines12060634

**Published:** 2024-06-07

**Authors:** Irit Nachtigall, Stefan Kwast, Sven Hohenstein, Sebastian König, Phi Long Dang, Johannes Leiner, Nicola Giesen, Benjamin Thomas Schleenvoigt, Marzia Bonsignore, Andreas Bollmann, Ralf Kuhlen, Fungwe Jah

**Affiliations:** 1 Department of Infectious Diseases and Infection Prevention, Helios Hospital Emil-von-Behring, 14165 Berlin, Germany; irit.nachtigall@helios-gesundheit.de; 2 Medical School Berlin, Chair of Infectiology and Immunology, 14197 Berlin, Germany; 3 Helios Health Institute, Real World Evidence and Health Technology Assessment, 13125 Berlin, Germany; sven.hohenstein@helios-health-institute.com (S.H.); sebastian.koenig@helios-gesundheit.de (S.K.); johannes.leiner@helios-gesundheit.de (J.L.);; 4 Department of Electrophysiology, Heart Center Leipzig, University of Leipzig, 04289 Leipzig, Germany; 5 Market Access, AstraZeneca, 22763 Hamburg, Germany; 6 Department of Hematology, Oncology and Palliative Care, Robert Bosch Hospital, 70376 Stuttgart, Germany; 7 Institute of Infectious Diseases and Infection Control, Jena University Hospital, 07743 Jena, Germany; 8 Center for Clinical and Translational Research, Helios Universitätsklinikum Wuppertal, University of Witten/Herdecke, 42283 Wuppertal, Germany; 9 Helios Health, 13125 Berlin, Germany; ralf.kuhlen@helios-health.com; 10 Medical Affairs, AstraZeneca, 22763 Hamburg, Germany

**Keywords:** Omicron, immunocompromised, SARI, in-hospital mortality, mechanical ventilation, intensive care unit, COVID-19 and SARS-CoV2

## Abstract

Aims: Endemic SARS-CoV-2 infections still burden the healthcare system and represent a considerable threat to vulnerable patient cohorts, in particular immunocompromised (IC) patients. This study aimed to analyze the in-hospital outcome of IC patients with severe SARS-CoV-2 infection in Germany. Methods: This retrospective, observational study, analyzed administrative data from inpatient cases (*n* = 146,324) in 84 German Helios hospitals between 1 January 2022 and 31 December 2022 with regard to in-hospital outcome and health care burden in IC patients during the first 12 months of Omicron dominance. As the primary objective, in-hospital outcomes of patients with COVID-19-related severe acute respiratory infection (SARI) were analyzed by comparing patients with (*n* = 2037) and without IC diagnoses (*n* = 14,772). Secondary analyses were conducted on IC patients with (*n* = 2037) and without COVID-19-related SARI (*n* = 129,515). A severe in-hospital outcome as a composite endpoint was defined per the WHO definition if one of the following criteria were met: intensive care unit (ICU) treatment, mechanical ventilation (MV), or in-hospital death. Results: In total, 12% of COVID-related SARI cases were IC patients, accounting for 15% of ICU admissions, 15% of MV use, and 16% of deaths, resulting in a higher prevalence of severe in-hospital courses in IC patients developing COVID-19-related SARI compared to non-IC patients (Odds Ratio, OR = 1.4, *p* < 0.001), based on higher in-hospital mortality (OR = 1.4, *p* < 0.001), increased need for ICU treatment (OR = 1.3, *p* < 0.001) and mechanical ventilation (OR = 1.2, *p* < 0.001). Among IC patients, COVID-19-related SARI profoundly increased the risk for severe courses (OR = 4.0, *p* < 0.001). Conclusions: Our findings highlight the vulnerability of IC patients to severe COVID-19. The persistently high prevalence of severe outcomes in these patients in the Omicron era emphasizes the necessity for continuous in-hospital risk assessment and monitoring of IC patients.

## 1. Introduction

The global landscape of the COVID-19 pandemic has evolved significantly, transitioning from a state of emergent crisis to an enduring endemic phase [1,2]. Since January 2022, the SARS-CoV-2 Omicron variant and its subvariants have been dominant among SARS-CoV-2 infections in Germany [3,4]. Various measures, in particular the development of vaccines and mandated hygiene measures, have led to a reduction in mortality rates and in the health care burden for patients [2,4,5]. However, approximately 154,000 COVID-19-related deaths still occurred in Germany in 2022 despite a base immunization rate of 76.4% [6]. Therefore, identifying vulnerable patient populations for severe outcomes of COVID-19 is still of immediate interest [2]. Among the variables influencing the disease severity of SARS-CoV-2 infections, the immune competence of patients emerges as a critical determinant, as immunocompromised (IC) patients demonstrate increased susceptibility to severe courses of the disease [1,7,8,9,10,11]. An ongoing individual immunocompromised state for, but not limited to, SARS-CoV-2 could be explained by a multi-factorial genesis on humoral and/or cellular mediated immunity [8], such as reduced antibody concentrations after antigen exposure [12], less neutralization activity of antibodies [13] or an accelerated decline in immunity after vaccination [14]. Despite the preventive efficacy of (active) vaccination among non-IC patients, for patients with insufficient immune competence, the availability of alternative antiviral treatment options, such as antiviral agents and monoclonal antibodies (passive immunization), offer additional options for the prevention and treatment of aggravated COVID-19 cases. [15]. In addition, there is also evidence for an ongoing need for intensified prevention and treatment of vulnerable IC patients regarding hospitalization [2,16]. In the Omicron era, the INFORM study revealed, for broadly defined IC patients in the UK, a base immunization rate of 93.6% and a rate of 84.5% for at least three active vaccinations, yet these patients still experience a confounder-adjusted 2.16 times higher risk of hospitalization [1]. Certain IC patient sub-cohorts such as solid organ transplant patients, experienced up to 14-fold increased risks regarding severe outcomes. Further, depending on the definition of IC patients, at least 4% of the population of Germany can be classified as immunocompromised [11], leading to an anticipated insufficient vaccination response of approximately 2% of the German population (1.5 million individuals) [17]. Current data on the in-hospital outcome for German cohorts, including hospitalized IC patients, are still rare [4,18], especially for patient populations with critical clinical courses who present with severe acute respiratory infections (SARI) related to infections with the endemic SARS-CoV-2 Omicron subtype.

This retrospective, cross-sectional, observational real-world data analysis within a German hospital cohort aims to evaluate the in-hospital course of IC patients infected during a period dominated by SARS-CoV-2 Omicron subtypes. Therefore, the primary objective of our study is the in-hospital outcome for a composite endpoint of severe courses in patients with COVID-19-related SARI, stratified for immune status. Additionally, the impact of SARS-CoV-2 infections within the IC cohort will be systematically evaluated as a secondary objective.

## 2. Methods

Data collection: In this retrospective, observational study, we analyzed administrative data from 84 hospitals (primary to tertiary care centers and university hospitals) within the national Helios group in Germany. The main diagnosis and comorbidities were identified according to the International Statistical Classification of Diseases and Related Health Problems (ICD-10-GM [German Modification]) [19]. In-hospital cases of pre-defined cohorts undergoing full inpatient treatment and admitted between 1 January 2022 and 31 December 2022 were included.

For the primary objective, we analyzed patient cohorts with COVID-19-related SARI with and without IC-related diagnoses. For contextualization, as a secondary objective, we analyzed IC patients with and without COVID-19-related SARI. The sub-cohorts of IC patients without COVID-19-related SARI also excluded COVID-19 patients without SARI to reduce confounding. The presence of SARS-CoV-2 infection was determined by laboratory-confirmed infection using the ICD-10-GM code U07.1. Comorbidities were classified by ICD-10-GM based Elixhauser comorbidity score with the Agency for Healthcare Research and Quality (AHRQ) algorithm [20,21]. SARI was defined in our claims dataset per the method of SARI surveillance if any of the ICD-10 diagnosis codes from ICD J09-J22 were coded, including ICD J09-J18 for influenza and pneumonia, ICD J20 for acute bronchitis, ICD J21 for acute bronchiolitis, and ICD J22 for acute lower respiratory tract infection [4,22,23].

IC patients were defined by the ICD-10 diagnosis claims database if at least one diagnosis met the classification of COVID-19-related broadly defined IC patients [16,24]. ICD-10 diagnoses will be coded during inpatient stay, if it is of significant relevance to the inpatient treatment. These ICD-10-based defined IC patients are assessed as clinically moderate to severe vulnerable conditions [10], including solid tumors, solid organ transplants, end-stage kidney disease, HIV infection, congenital immunodeficiency, autoimmune diseases, hematological malignancy, and end-stage chronic liver diseases. Detailed ICD-10-based definitions for IC-cohorts are provided in Supplementary Material Table S1. 

In-hospital medical procedures were used to describe the extent of in-hospital care and were identified by coded operations and procedures codes (OPS) [25]. Severe in-hospital cases were classified per the WHO stages of COVID-19 [26], including the following stratification: treatment in an intensive care unit (ICU) (OPS-codes 8-980, 8-98f or length of stay on ICU > 0 days), mechanical ventilation (OPS-codes 8-70x, 8-71x or length of mechanical ventilation > 0 days) or in-hospital death (determined by hospital discharge type in administrative data). Cases discharged to another hospital were excluded for analysis of mortality. In addition, the healthcare burden was analyzed by length of stay and hospitalization costs based on the total cost of healthcare insurance. 

The study followed the STROBE guidelines for observational analysis [27]. Patients’ data were stored in a double-pseudonymized form, and the use of the data was approved by the local ethics committee of the University of Leipzig (AZ490/20-ek) and the Helios Kliniken GmbH data protection authority. Considering the retrospective analysis of double-pseudonymized administrative routine clinical data, individual informed consent was neither applicable nor obtained. 

Statistical analysis: Administrative data were extracted from QlikView (QlikTech, Radnor, PA, USA), and all analyses were performed within the R statistical computing environment. Baseline characteristics were presented as proportions (for categorical variables) and either means with standard deviations (for continuous variables) or medians with interquartile range (IQR). To describe the patient characteristics of the cohorts and comorbidities, we employed χ2-tests for categorical variables and a two-sample *t*-test for numerical variables. We used logistic generalized linear mixed models (GLMM; [28]) with a logit link function to compare the proportions of selected treatments and outcomes in the different cohorts. In all mixed models, we specified varying intercepts for the random factor (hospitals). The numeric variables, length of stay, and hospitalization costs were analyzed via linear mixed models (LMM). Because these variables were positively skewed, we transformed them via inverse hyperbolic sine to roughly approximate normal distributions. We apply a two-tailed 5% error criterion for significance for all tests.

Multivariable analyses of outcomes were performed via logistic GLMM and LMM. Categorical variables entered the analyses as simple-coding contrasts, while the continuous variable age was centered at its mean. All continuous variables were scaled to unit variance.

## 3. Results

**Figure 1:**
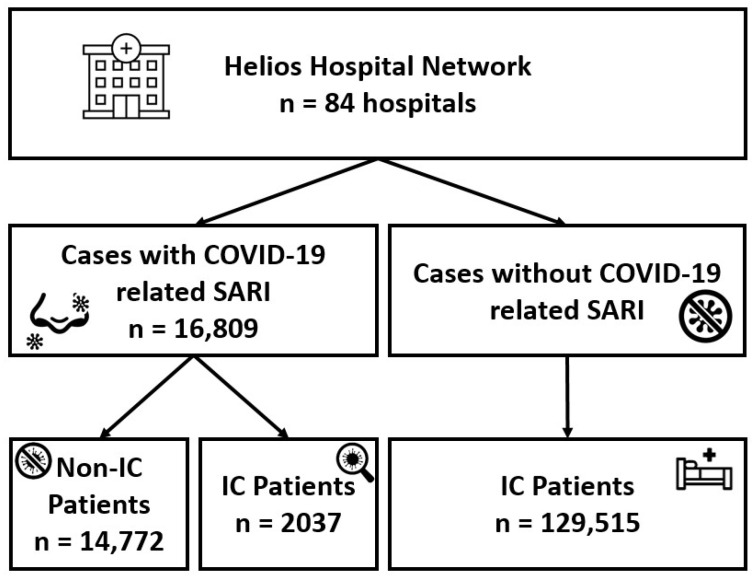
Flow chart of cohort extraction. IC = immunocompromised; SARI = severe acute respiratory illness.

**Figure 2:**
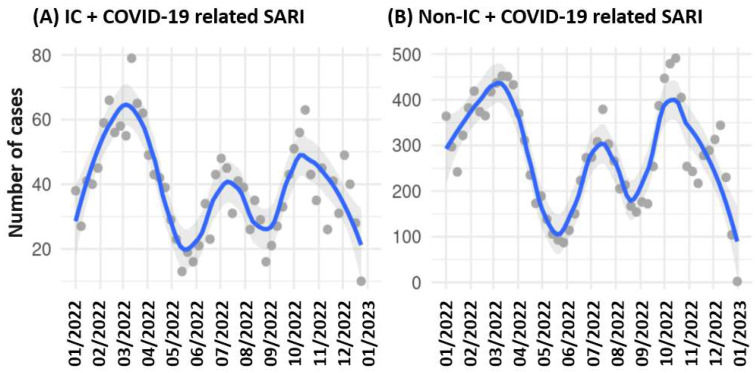
Weekly case numbers for COVID-19-related SARICOVID-19-related SARI cases of (**A**) IC patients and (**B**) non-IC patients. Blue: LOESS curve (locally estimated scatterplot smoothing). Gray: 95% confidence interval, dots: case number per week.

Out of 87 hospitals in the German-wide Helios hospital network, 84 contributed (*n* = 146,324) cases for the retrospective data analyses. A flow chart of cohort identification is presented in Figure 1. A total of 16,809 cases were identified as COVID-19-related SARI cases. Of these, 14,772 (87.9%) cases were non-IC patients, and 2037 (12.1%) were IC patients. Additionally, 129,515 IC hospitalized patients were identified without COVID-19 and SARI. We have not further specified cases without COVID-19 and without an underlying IC disease, as this would include all patients in the hospital network and is not related to the research question. Throughout the analyzed period, we observed a fluctuating trend in hospitalization with three major waves of SARS-CoV-2 infection in 2022: as illustrated in Figure 1 for IC and non-IC patients, the first wave of infection ran from January to April, the second from May to August and the third from September to December (Figure 2). 

### 3.1. Baseline Characteristics of Patients with COVID-19-Related SARI, with and without IC 

Baseline characteristics for COVID-19-related SARI cases are presented in Table 1. The IC patient cohort was significantly younger (−1.9 years, *p* < 0.001), had significantly more comorbidities (+9.3 Elixhauser comorbidity score, *p* < 0.01), and included 3% more men (*p* = 0.015). Highest comorbidity incidences in COVID-19-related SARI cases were fluid and electrolyte disorders (52% in non-IC, 54% in IC, *p* = 0.214), uncomplicated arterial hypertension (45% in non-IC, 42% in IC, *p* = 0.007), renal failure (35% in non-IC, 39% in IC, *p* < 0.001), cardiac arrhythmias (34% in non-IC, 28% in IC, *p* < 0.001) and congestive heart failure (31% in non-IC, 29% in IC, *p* = 0.050). A list of incidences for Elixhauser comorbidity clustered diagnoses is provided in the Supplementary Material Table S2.

### 3.2. In-Hospital Outcome for Patients with COVID-19-Related SARI with and without IC 

The primary objective of evaluating the severe in-hospital course for COVID-19-related SARI revealed that IC patients exhibited a significantly higher prevalence rate of 42%, compared to 32% in patients without an IC diagnosis, resulting in an odds ratio (OR) of 1.4 (*p* < 0.001, see Table 2). This increased risk was based on an increased prevalence in all severity stratifications: ICU treatment (OR = 1.3, *p* < 0.001), increased prevalence of mechanical ventilation (OR = 1.2, *p* = 0.020) and increased in-hospital mortality (OR = 1.5, *p* < 0.001). The length of hospital stay was prolonged by 4.6 days (41%) for IC vs. non-IC patients affected with COVID-19-related SARI and the cost of hospitalization was 49% higher in these cases (IC patients: vs. non-IC patients: 12,611.90€ vs. 8318.80€, *p* < 0.001). Table 2 summarizes the in-hospital outcomes for the primary research objective.

**Figure 3:**
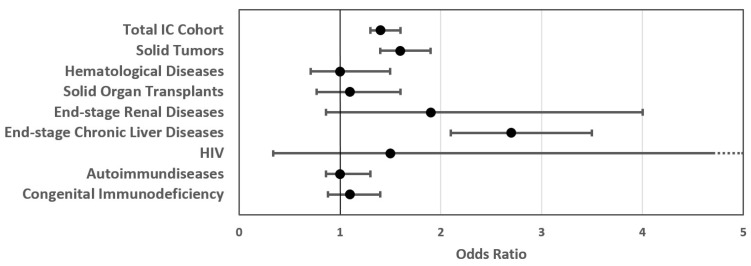
Odds ratios for severe outcomes in the total IC patient cohort and IC patient sub-cohorts compared to the non-IC patient cohort.

In the diagnosis-stratified sub-cohorts of IC patients with COVID-19-related SARI, we found different outcomes for the prevalence of severe in-hospital courses compared to non-IC patients (Table 2). The risk for severe outcomes (composite endpoint) of IC sub-cohorts is presented in Figure 3. Across all IC sub-cohorts, there was a trend towards a higher prevalence of severe outcomes, either within the composite endpoint itself or within its specific sub-domains. A significantly higher prevalence of ICU treatment was observed for IC patients with solid organ transplants, end-stage chronic liver diseases, and autoimmune diseases. ICU treatment with mechanical ventilation was at a significantly higher prevalence for patients with end-stage chronic liver diseases. Patients with solid tumors and end-stage chronic liver diseases showed a significantly increased in-hospital mortality compared to non-IC patients. 

### 3.3. Baseline Characteristics of Hospitalized IC Patients, with and without COVID-19-Related SARI 

A total of *n* = 129,515 hospitalizations of IC patients and *n* = 2037 cases of IC patients with COVID-19-related SARI occurred during the analyzed Omicron-dominant period per our IC definition. IC patients had a mean hospitalization case rate per month of *n* = 10,793 during the defined Omicron period. 

In comparison to IC patients with COVID-19-related SARI, the IC patient cohort without COVID-19-related SARI was significantly younger (66.3 vs. 71.2 years, *p* < 0.001), had 4% fewer males (55% vs. 59% *p* < 0.001) and had a lower Elixhauser Comorbidity score (15.3 vs. 21.4 *p* < 0.001). Patient characteristics for total IC patients are shown in Table 3.

Main comorbidities were less prevalent in IC patients compared to IC patients with COVID-19-related SARI, except for uncomplicated arterial hypertension. The most prevalent comorbidities were fluid and electrolyte disorders (18% in IC, 54% in IC SARI, *p* < 0.001), uncomplicated arterial hypertension (44% in IC, 42% in IC SARI, *p* = 0.104), renal failure (22% in IC, 39% in IC SARI, *p* < 0.001), cardiac arrhythmias (15% in IC, 28% in IC SARI, *p* < 0.001) and congestive heart failure (12% in IC, 29% in IC SARI, *p* < 0.001). The data for comorbidities within the total IC cohort and stratified for IC sub-cohorts are provided in the Supplementary Table S2.

### 3.4. In-Hospital Outcome for IC Patients with and without COVID-19-Related SARI

**Figure 4:**
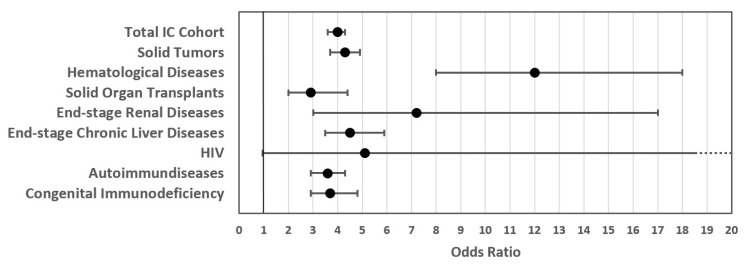
Odds ratios for severe outcomes in the total IC patient cohort and IC patient sub-cohorts with COVID-19-related SARI compared to the IC patient cohort without SARI.

In-hospital outcomes for the cohort of IC patients with and without COVID-19-related SARI are shown in Table 4. Results stratified by diagnosis-based IC sub-cohorts are shown in Figure 4 for the composite endpoint of severe outcomes, and results for subdomains ICU treatment, mechanical ventilation, and mortality are provided in the Supplementary Material Table S4. Analysis of the secondary objective for IC patients with and without COVID-19-related SARI showed an odds ratio of 4.0 for the severe course during hospitalization for IC patients with COVID-19-related SARI (*p* < 0.001). Severe courses were seen in 42% of IC patients with COVID-19-related SARI compared to 14% in the total IC cohort. More specifically, the odds ratios for IC patients with COVID-19-related SARI were 3.1 for ICU treatment (*p* < 0.001), 10.0 for mechanical ventilation (*p* < 0.001), and 6.0 for in-hospital mortality (*p* < 0.001). These findings were confirmed in all IC sub-cohorts stratified by IC diagnosis. Notably, patients with hematologic disease had the highest risk of severe in-hospital outcomes for COVID-19-related SARI compared to the total IC cohort (OR for severe course = 12.0, *p* < 0.001).

Multivariable analyses for in-hospital outcomes in IC patients identified COVID-19-related SARI as the variable with the highest OR of 3.23 for the composite endpoint of severe outcomes, as well as sex (male, OR = 1.07, *p* < 0.01), age (OR = 1.07, *p* < 0.001), and Elixhauser comorbidity score (OR = 2.67, *p* < 0.01), showing significant association with severe outcomes. These findings from the multivariable analysis showed that significantly increased OR for COVID-19-related SARI on hospital outcomes in IC patients were concordant for ICU treatment (OR = 2.59, *p* < 0.01), mechanical ventilation (OR = 8.07, *p* < 0.001) and in-hospital mortality (OR = 4.44, *p* < 0.001). 

An increased in-hospital length of stay was associated with male sex, age, Elixhauser comorbidity score, and the prevalence of COVID-19-related SARI. Cost for hospitalization showed an association with the male sex, COVID-19-related SARI, and Elixhauser comorbidity scores. Table 5 summarizes the results of multivariable analyses for in-hospital courses of IC patients.

## 4. Discussion

The global course of the COVID-19 pandemic has undergone a paradigm shift, transitioning from an acute pandemic crisis to a protracted endemic state. In this dynamic development, a nuanced understanding of the disease’s impact on various patient cohorts, especially those with reduced immune competence, remains important [2]. Our retrospective, cross-sectional observational analysis evaluated in-hospital outcomes for COVID-19-related SARI within a multi-centric German in-hospital cohort, providing evidence on the risk stratification of severe courses in IC patients during the emergence of the Omicron variant.

The temporal trend analysis for 2022 revealed recurrent waves of SARS-CoV-2 infection, with a congruent trend in IC and non-IC patients, since peak infection rates occurred in the same months. This observation suggests that the infection rates of IC patients are driven by the general trend of infection rates in Germany, a pattern consistent with findings reported in the United States [10]. Ketkar et al. demonstrated a close link in the incidence of infection between non-IC and IC patients over the course of the pandemic until 2022 in the US [29]. Preventive measures to protect all patients can also indirectly protect IC patients, as this pattern was repetitively shown over different parts of the pandemic and in different countries.

The characteristics of our IC cohort showed a lower mean age, a higher number of comorbidities, and a slightly higher proportion of men compared to non-IC patients, aligning with other studies in hospitalized IC cohorts [30,31]. Notably, the most prevalent comorbidities in both cohorts included fluid and electrolyte disorders, uncomplicated arterial hypertension, renal failure, cardiac arrhythmias, and congestive heart failure. These latter four comorbidities were also found as most prevalent in IC patients by Singson et al. in a multi-state observational study conducted in the United States [31]. The authors reported a proportion of patients with three or more comorbidities of 69.3% but did not provide a mean number or index for total comorbidities. Our IC cohort also showed a high proportion for comorbidities of 85% for at least five points on the Elixhauser comorbidity index. 

Regarding the primary objective of our study, which involved analyzing severe in-hospital outcomes for patients with COVID-19-related SARI based on immune status, we provided evidence of persistently elevated rates for severe courses in IC individuals during the Omicron period. An odds ratio of 1.4 demonstrated an increased risk for IC patients compared to non-IC patients with COVID-19-related SARI. Nevejan et al. reported a higher OR of 2.1 for severe outcomes in a more narrow defined in-hospital IC cohort across a small number of hospitals [30]. Our cohort reflects a broader perspective of in-hospital care, encompassing primary, secondary, and tertiary care providers, which may explain the lower risk for severe outcomes. The increased risk for severe outcomes is further supported by increased rates of ICU treatments, mechanical ventilation, and in-hospital mortality. The odds ratios for ICU treatment and death were 1.3 and 1.5, respectively, which are consistent with the odds ratios reported by Singson et al., who reported ORs of 1.4 for ICU treatment and 1.9 for mortality in vaccinated IC patients [31]. Additionally, a meta-analysis conducted by Khoury et al. reported risk ratios of 1.9 for overall mortality among cancer patients [32]. Similarly, in our study, patients with solid tumors or end-stage chronic liver disease had significantly increased risks for the severe courses, consistent with findings from previous studies [32,33]. Although patients with hematologic diseases and end-stage chronic kidney disease also showed a trend towards increased risk for severe outcomes, this did not reach significance, probably due to the smaller sample size for these less prevalent diseases. However, the trend is consistent with data provided from other observational studies [1,34]. 

Yin et al. also reported an increased hospital length of stay and mortality for IC patients, showing a comparable increase in mortality of 50% [35]. Our data also confirm the mortality rate (OR 1.44) for hospitalized patients found by Turtle et al. in the United Kingdom [36], although the majority of cases considered here were the early variants of SARS-CoV-2. Thus, compared to our data, the risk of IC patients is still relatively present compared to non-IC patients. Invasive ventilation showed heterogeneous results in specific IC subgroups in sub-analyses provided by Turtle et al. (2023), for example lower rates in cancer patients. While we found a clear trend toward higher rates of mechanical ventilation, not all sub-cohorts showed a statistically significant increased risk. Prolonged hospital stays and increased hospitalization costs underscore the increased healthcare burden in IC individuals infected with the Omicron variant. The EPOCH study found that hospitalization costs were, on average, 2.46 times higher for their IC composite cohort when comparing pre vs. post-COVID-19 hospitalization costs [10]. In our analysis, hospitalization costs for IC patients with COVID-19 were 2.19 times higher compared to IC patients without COVID-19, confirming the findings of the EPOCH study. In addition, hospitalization costs at the onset of the COVD-19 pandemic in the year 2020 were at comparable levels for IC patients, as we found for our 2022 cohort [18]. 

Age and sex (male) were identified as predictors for severe in-hospital outcomes in our COVID-19-related SARI cohort, consistent with findings reported by Nevejan et al. for IC patients and by Bonsignore et al. for non-IC patients [30,37]. Among patients who developed a COVID-19-related SARI, IC patients showed increased risk for severe in-hospital courses. 

Therefore, while active vaccination reduces susceptibility to severe disease and our clinical treatment practices continue to accumulate knowledge, our data support the recommendation to provide continued special attention to vulnerable groups [2,15,38].

The secondary objective, analyzing the impact of COVID-19-related SARI in IC cohorts, showed a fourfold increased risk of severe outcomes in IC patients with COVID-19-related SARI compared to IC patients without COVID-19-related SARI. Multivariable analyses confirmed this increased risk for severe outcomes, even after correcting for age, male sex, and comorbidity scores. SARS-CoV-2 infection and SARI also showed a significant effect when analyzed separately by multivariate analysis (SARS-CoV-2 infection OR = 1.31, *p* < 0.001; SARI OR = 3.23, *p* < 0.001). Therefore, SARS-CoV-2 infections and the development of COVID-19-related SARI have a significant impact on the in-hospital outcome of IC patients. Increased risks for severe in-hospital outcomes were not homogenous between all IC sub-cohorts. However, despite a small cohort of HIV patients, all IC sub-cohorts showed a pronounced increased risk for severe in-hospital outcome with OR ranging from 2.9 (solid organ transplants) to 12.0 (hematological diseases). This finding underlines the need for diagnosis-specific patient risk evaluations among hospitalized patients in the IC sub-cohort.

Our study results highlight both clinical and public health implications. The particular vulnerability of immunocompromised individuals requires an increased awareness during in-hospital treatments and underlines the importance of strategies like proactive vaccination initiatives [39], preventive measures like pre-exposure prophylaxis (PREP) [40], and general public hygiene rules.

Different active vaccines may also trigger different immune responses [41]. Comorbidities and genetic predispositions also influence immunocompetence and symptom severity [42]. Increased vaccination regimens did not necessarily result in improved humoral or cellular immune responses in IC patients [43]. Moreover, different vaccines were proven for different effectiveness in IC patients in observational and more real-world driven studies [44]. Further studies in IC patients could lead to specific recommendations in this context. In addition, antiviral therapies with passive immunization, for example, have been recommended in the German COVID-19 guidelines to be considered in individual cases for patients with a reduced immune response [45]. Our data suggest that diagnoses and comorbidities can be used to specifically identify vulnerable patients and to protect patients in the context of hospitalizations and medical interventions. In summary, this study contributes additional evidence regarding the impact of the Omicron variant on immunocompromised cohorts. Analysis of in-hospital outcomes, differential risk factors, and disease-specific comorbidities contributes to a refined understanding of the determinants that drive disease severity in this vulnerable population. Further research efforts are necessary to continually refine our findings and identify and evaluate targeted interventions to optimize outcomes in immunocompromised individuals affected by SARS-CoV-2 infections.

## 5. Limitations

This study is based on administrative data primarily collected for remuneration purposes rather than research interests, which potentially could affect the encoded information. The quality of our results depends to a large extent on the accurate encoding of procedures and diagnoses at hospital discharge. Nevertheless, claims data are continuously monitored and checked at the hospital level by medical coding personnel and health insurance providers, and were introduced in 2022 as a surveillance method in Germany by the federal Robert Koch Institute [46]. Moreover, reimbursement companies and health insurance providers continually evaluate administrative hospital discharge data to ensure data accuracy. 

In addition, the ICD-10-based assessment of IC states did not include all patients undergoing specific therapies for immunosuppression due to the unavailability of information on medication. Furthermore, it is also not possible to estimate the severity of immune suppression. Therefore, an underestimation of IC prevalence is possible in our cohort. In addition, our cohorts of diagnosis-based IC patients varied in sample size, which led to tendencies for severe outcomes without reaching significant levels due to higher confidence intervals.

Due to the data structure and data protection regulations, especially case pseudonymization, linking patient data between different hospitals was not possible. This limitation implies the possibility of double-counting specific patients hospitalized multiple times or due to multiple SARS-CoV2 infections within our data periods. The claims-based analysis did not include vaccination information. However, since it is known that IC patients have higher vaccination rates than non-IC patients, our risk increases tend to be conservative and thus ultimately reflect the clinical real-world outcomes.

## 6. Conclusions

IC patients still have an increased risk for severe in-hospital courses by developing COVID-19-related SARI. Age, male sex, and a high number of comorbidities are predictors for severe outcomes and should be considered potential risk factors within hospital care. This study highlights the importance of prevention by active vaccination or passive PREP prior to hospitalization. Hospitalized patients should be closely monitored to reduce the risks associated for ICU treatment, mechanical ventilation and death by providing early antiviral therapy, treating comorbidities, and symptoms. Ongoing evaluation and intensified care of vulnerable patients may contribute to reduced severe outcomes in the future.

## Figures and Tables

**Table 1:** Baseline characteristics of SARS-CoV-2-related SARI patients, stratified for non-IC patients, IC patients and IC patient sub-cohorts.

Characteristic ^1^	Non-IC COVID-19-Related SARICOVID-19-Related SARI	IC COVID-19-Related SARICOVID-19-Related SARI	*p*-Value ^2^	Solid Tumors with COVID-19-Related SARICOVID-19-Related SARI	*p*-Value ^2^	Hematological Diseases with COVID-19-Related SARICOVID-19-Related SARI	*p*-Value ^2^	Solid Organ Transplants with COVID-19-Related SARICOVID-19-Related SARI	*p*-Value ^2^	End-Stage Renal Disease with COVID-19-Related SARICOVID-19-Related SARI	*p*-Value ^2^	End-Stage Chronic Liver Diseases with COVID-19-Related SARICOVID-19-Related SARI	*p*-Value ^2^	HIV with COVID-19-Related SARICOVID-19-Related SARI	*p*-Value ^2^	Autoimmune Diseases with COVID-19-Related SARICOVID-19-Related SARI	*p*-Value ^2^	Congenital Immunodeficiency with COVID-19-Related SARICOVID-19-Related SARI	*p*-Value ^2^
Case numbers	N = 14,772	N = 2037		N = 1000		N = 133		N = 138		N = 27		N = 243		N = 7		N = 494		N = 347	
Age (years)	73.1 (19.7)	71.2 (13.1)	<0.001	73.6 (11.1)	0.182	70.0 (13.6)	0.010	61.6 (13.1)	<0.001	74.1 (11.5)	0.665	67.4 (12.3)	<0.001	46.6 (6.3)	<0.001	71.3 (14.5)	0.007	66.9 (15.1)	<0.001
Age group			<0.001		<0.001		<0.001		<0.001		0.695		<0.001		<0.001		<0.001		<0.001
≤59 years	2310 (16%)	360 (18%)		106 (11%)		25 (19%)		52 (38%)		4 (15%)		66 (27%)		7 (100%)		99 (20%)		98 (28%)	
60−69 years	1797 (12%)	470 (23%)		231 (23%)		34 (26%)		51 (37%)		5 (19%)		64 (26%)		0 (0%)		96 (19%)		83 (24%)	
70−79 years	3356 (23%)	574 (28%)		309 (31%)		39 (29%)		26 (19%)		7 (26%)		68 (28%)		0 (0%)		124 (25%)		92 (27%)	
≥80 years	7309 (49%)	633 (31%)		354 (35%)		35 (26%)		9 (6.5%)		11 (41%)		45 (19%)		0 (0%)		175 (35%)		74 (21%)	
Sex			0.015		<0.001		0.004		0.193		1.000		<0.001		0.238		<0.001		0.643
Male	8337 (56%)	1208 (59%)		644 (64%)		92 (69%)		86 (62%)		15 (56%)		169 (70%)		6 (86%)		208 (42%)		191 (55%)	
Female	6435 (44%)	829 (41%)		356 (36%)		41 (31%)		52 (38%)		12 (44%)		74 (30%)		1 (14%)		286 (58%)		156 (45%)	
Elixhauser comorbidity score	12.1 (10.4)	21.4 (13.5)	<0.001	27.2 (13.0)	<0.001	18.5 (13.2)	<0.001	15.1 (11.1)	0.002	17.6 (10.0)	0.009	24.5 (12.3)	<0.001	14.4 (12.1)	0.632	13.6 (10.8)	0.002	15.8 (12.8)	<0.001

^1^ Data are presented as mean (SD) or n (%); ^2^ Welch Two Sample *t*-test/Pearson’s Chi-squared test compared against No IC SARI SARS-CoV-2 patient cohort.

**Table 2:** In-hospital outcomes for patients with COVID-19-related SARI.

	Non-IC with COVID-19-Related SARI, N = 14,772	IC with COVID-19-Related SARI, N = 2037	Odds Ratio [95% CI]	*p*-Value	Solid Tumors with COVID-19-Related SARI, N = 1000	Odds Ratio [95% CI]	*p*-Value	Hematological Diseases with COVID-19-Related SARI, N = 133	Odds Ratio [95% CI]	*p*-Value	Solid Organ Transplants with COVID-19-Related SARI, N = 138	Odds Ratio [95% CI]	*p*-Value	End-Stage Renal Disease with COVID-19-Related SARI, N = 27	Odds Ratio [95% CI]	*p*-Value
Intensive care (n (%))	3273 (22%)	577 (28%)	1.3 [1.2–1.4]	<0.001	249 (25%)	1.1 [0.9–1.3]	0.272	35 (26%)	1.0 [0.7–1.5]	0.910	44 (32%)	1.5 [1.0–2.2]	0.035	9 (33%)	1.5 [0.7–3.5]	0.321
Mechanical ventilation (n (%))	1984 (13%)	336 (16%)	1.2 [1.0–1.3]	0.020	145 (15%)	1.0 [0.8–1.2]	0.979	24 (18%)	1.1 [0.7–1.7]	0.721	26 (19%)	1.4 [0.9–2.1]	0.178	7 (26%)	1.7 [0.7–4.1]	0.243
Severe course (n (%))	4693 (32%)	840 (42%)	1.4 [1.3–1.6]	<0.001	439 (45%)	1.6 [1.4–1.9]	<0.001	50 (38%)	1.0 [0.7–1.5]	0.933	47 (37%)	1.1 [0.8–1.6]	0.582	14 (52%)	1.9 [0.9–4.0]	0.114
N/A	312	50			27			1			10			0		
In-hospital mortality (n (%))	2331 (17%)	458 (24%)	1.5 [1.4–1.7]	<0.001	275 (29%)	2.0 [1.7–2.3]	<0.001	33 (25%)	1.5 [1.0–2.2]	0.059	17 (15%)	0.8 [0.5–1.4]	0.446	6 (24%)	1.3 [0.5–3.4]	0.536
N/A	693	120			52			3			21			2		
Length of stay (d)	11.2 (12.6)	15.8 (15.3)		<0.001	17.0 (16.0)		<0.001	19.8 (17.1)		<0.001	11.8 (12.2)		0.632	18.3 (17.1)		0.028
Costs (€)	8318.8 (14,958.9)	12,377.7 (18,772.4)		<0.001	12,611.9 (19,733.9)		<0.001	17,857.8 (20,548.9)		<0.001	11,232.6 (13,757.8)		<0.001	16,688.0 (30,440.2)		0.007
**End-Stage Chronic Liver Disease with COVID-19-related SARI, N = 243**		**Odds Ratio [95% CI]**	***p*-Value**	**HIV with COVID-19-related SARI, N = 7**		**Odds Ratio [95% CI]**	***p*-Value**	**Autoimmune Disease with COVID-19-related SARI, N = 494**		**Odds Ratio [95% CI]**	***p*-Value**	**Congenital Immunodeficiency with COVID-19-related SARI, N = 347**			**Odds Ratio [95% CI]**	***p*-Value**
103 (42%)		2.5 [1.9–3.3]	<0.001	3 (43%)		2.8 [0.6–13.0]	0.193	132 (27%)		1.3 [1.0–1.5]	0.031	94 (27%)			1.2 [1.0–1.6]	0.085
53 (22%)		1.7 [1.2–2.3]	0.002	2 (29%)		2.7 [0.5–15.0]	0.253	79 (16%)		1.2 [0.9–1.5]	0.179	59 (17%)			1.3 [0.9–1.7]	0.129
137 (57%)		2.7 [2.1–3.5]	<0.001	3 (43%)		1.5 [0.3–7]	0.575	164 (34%)		1.0 [0.9–1.3]	0.695	120 (36%)			1.1 [0.9–1.4]	0.389
4				0				7				9				
76 (33%)		2.5 [1.9–3.3]	<0.001	1 (14%)		0.8 [0.1–6.5]	0.820	71 (15%)		0.9 [0.7–1.1]	0.327	58 (18%)			1.1 [0.8–1.4]	0.639
16				0				24				20				
19.5 (18.2)			<0.001	19.3 (18.0)			0.072	12.7 (12.9)			<0.001	13.3 (12.8)				<0.001
15,673.2 (20,015.1)			<0.001	19,703.3 (22,122.9)			0.014	9835.0 (16,659.7)			<0.001	11,488.5 (18,718.4)				<0.001

**Table 3:** Characteristics of IC patients, IC patients with COVID-19-related SARI, and IC patient sub-cohorts.

Characteristic ^1^		IC, N = 129,515		IC with COVID-19-Related SARI, N = 2037	*p*-Value ^2^		Solid Tumors, N = 92,376	Solid Tumors with COVID-19-Related SARI, N = 10,001	*p*-Value ^2^	Hematological Diseases, N = 5217	Hematological Diseases with COVID-19-Related SARI, N = 133	*p*-Value^2^	Solid Organ Transplants, N = 1842	Solid Organ Transplants with COVID-19-Related SARI, N = 138	*p*-Value ^2^	
Age (years)		66.3 (15.1)		71.2 (13.1)	<0.001		67.9 (13.7)	73.6 (11.1)	<0.001	56.3 (24.9)	70.0 (13.6)	<0.001	59.5 (14.9)	61.6 (13.1)	0.076	
Age group					<0.001				<0.001			<0.001			0.768	
≤59 years		35,067 (27%)		360 (18%)			21,316 (23%)	106 (11%)		2155 (41%)	25 (19%)		771 (42%)	52 (38%)		
60−69 years		35,529 (27%)		470 (23%)			26,017 (28%)	231 (23%)		1166 (22%)	34 (26%)		630 (34%)	51 (37%)		
70−79 years		33,218 (26%)		574 (28%)			25,419 (28%)	309 (31%)		1091 (21%)	39 (29%)		342 (19%)	26 (19%)		
≥80 years		25,701 (20%)		633 (31%)			19,624 (21%)	354 (35%)		805 (15%)	35 (26%)		99 (5.4%)	9 (6.5%)		
Sex					<0.001				<0.001			0.012			1.000	
Male		70,686 (55%)		1208 (59%)			52,865 (57%)	644 (64%)		3021 (58%)	92 (69%)		1151 (62%)	86 (62%)		
Female		58,827 (45%)		829 (41%)			39,510 (43%)	356 (36%)		2196 (42%)	41 (31%)		691 (38%)	52 (38%)		
N/A		2		0			1	0								
Elixhauser comorbidity score		15.3 (11.8)		21.4 (13.5)	<0.001		18.1 (11.4)	27.2 (13.0)	<0.001	11.5 (10.4)	18.5 (13.2)	<0.001	10.6 (9.5)	15.1 (11.1)	<0.001	
Elixhauser comorbidity index					<0.001				<0.001			0.014			<0.001	
<0		5933 (4.6%)		48 (2.4%)			252 (0.3%)	2 (0.2%)		107 (2.1%)	2 (1.5%)		110 (6.0%)	4 (2.9%)		
0		5157 (4.0%)		45 (2.2%)			40 (<0.1%)	0 (0%)		742 (14%)	7 (5.3%)		186 (10%)	2 (1.4%)		
1–4		8587 (6.6%)		69 (3.4%)			3701 (4.0%)	5 (0.5%)		298 (5.7%)	5 (3.8%)		149 (8.1%)	7 (5.1%)		
≥5		109,838 (85%)		1875 (92%)			88,383 (96%)	993 (99%)		4070 (78%)	119 (89%)		1397 (76%)	125 (91%)		
**End-stage renal disease**				**End-stage chronic liver diseases**			**HIV**			**Autoimmune diseases**			**Congenital immunodeficiency**			
End-stage renal disease, N = 709	**End-stage renal disease with COVID-19-related SARI, N = 27**		***p*-value ^2^**	**End-stage chronic liver diseases, N = 11,040**	**End-stage chronic liver diseases with COVID-19-related SARI, N = 243**	***p*-value ^2^**	**HIV, N = 176**	**HIV with COVID-19-related SARI, N = 7**	***p*-value ^2^**	**Autoimmune diseases, N = 20,976**	**Autoimmune diseases with COVID-19-related SARI, N = 494**	***p*-value ^2^**	**Congenital immunodeficiency, N = 6508**	**Congenital immunodeficiency with COVID-19-related SARI, N = 347**		***p*-value ^2^**
69.0 (13.7)	74.1 (11.5)		0.034	65.6 (12.2)	67.4 (12.3)	0.022	50.9 (13.8)	46.6 (6.3)	0.138	63.8 (16.0)	71.3 (14.5)	<0.001	52.2 (25.7)	66.9 (15.1)		<0.001
			0.443			0.085			0.473			<0.001				<0.001
161 (23%)	4 (15%)			3316 (30%)	66 (27%)		129 (73%)	7 (100%)		7571 (36%)	99 (20%)		3296 (51%)	98 (28%)		
158 (22%)	5 (19%)			3445 (31%)	64 (26%)		24 (14%)	0 (0%)		5109 (24%)	96 (19%)		1346 (21%)	83 (24%)		
198 (28%)	7 (26%)			2701 (24%)	68 (28%)		18 (10%)	0 (0%)		4422 (21%)	124 (25%)		1042 (16%)	92 (27%)		
192 (27%)	11 (41%)			1578 (14%)	45 (19%)		5 (2.8%)	0 (0%)		3874 (18%)	175 (35%)		824 (13%)	74 (21%)		
			0.809			0.176			1.000			0.013				0.205
424 (60%)	15 (56%)			7194 (65%)	169 (70%)		143 (81%)	6 (86%)		7665 (37%)	208 (42%)		3345 (51%)	191 (55%)		
285 (40%)	12 (44%)			3846 (35%)	74 (30%)		33 (19%)	1 (14%)		13,310 (63%)	286 (58%)		3163 (49%)	156 (45%)		
										1	0					
13.5 (9.8)	17.6 (10.0)		0.047	15.6 (11.8)	24.5 (12.3)	<0.001	6.4 (9.6)	14.4 (12.1)	0.131	6.1 (9.4)	13.6 (10.8)	<0.001	8.7 (11.4)	15.8 (12.8)		<0.001
			0.245			<0.001			0.193			<0.001				<0.001
10 (1.4%)	0 (0%)			453 (4.1%)	2 (0.8%)		34 (19%)	0 (0%)		4747 (23%)	31 (6.3%)		842 (13%)	16 (4.6%)		
26 (3.7%)	0 (0%)			109 (1.0%)	0 (0%)		38 (22%)	0 (0%)		3672 (18%)	28 (5.7%)		1453 (22%)	26 (7.5%)		
59 (8.3%)	0 (0%)			2048 (19%)	11 (4.5%)		14 (8.0%)	1 (14%)		2323 (11%)	34 (6.9%)		592 (9.1%)	22 (6.3%)		
614 (87%)	27 (100%)			8430 (76%)	230 (95%)		90 (51%)	6 (86%)		10,234 (49%)	401 (81%)		3621 (56%)	283 (82%)		

^1^ Data are presented as mean (SD) or n (%); ^2^ Welch Two Sample *t*-test/Pearson’s Chi-squared test compared against No IC SARI SARS-CoV-2 patient cohort.

**Table 4:** In-hospital outcomes for IC patients, with and without SARS-CoV-2-related SARI.

Characteristic	(Total)				
	IC without COVID-19-Related SARI, N = 129,515	IC with COVID-19-Related SARI, N = 2037	Odds Ratio	95% CI	*p*-Value
Intensive care	13,310 (10%)	577 (28%)	3.1	2.8, 3.4	<0.001
Mechanical ventilation	2372 (1.8%)	336 (16%)	10	8.9, 11	<0.001
Severe course	18,090 (14%)	840 (42%)	4.0	3.6, 4.3	<0.001
N/A	3175	50			
In-hospital mortality	5836 (4.7%)	458 (24%)	6.0	5.3, 6.6	<0.001
N/A	4352	120			
Length of stay (d)	6.1 (7.2)	15.8 (15.3)			<0.001
Costs (€)	5645.3 (6877.4)	12,377.7 (18,772.4)			<0.001

**Table 5:** Results of multivariate analyses in IC cases for in-hospital outcomes.

	ICU Treatment		Mechanical Ventilation		In-Hospital Mortality		Severe Courses		Length of Stay		Costs of Hospitalization	
Variable	OR (95% CI)	*p * Value	OR (95% CI)	*p * Value	OR (95% CI)	*p * Value	OR (95% CI)	*p * Value	Coefficient (95% CI)	*p * Value	Coefficient (95% CI)	*p * Value
Male sex	1.10 (1.06–1.14)	<0.001	1.14 (1.05–1.23)	0.001	1.04 (0.98–1.10)	0.167	1.07 (1.04–1.11)	<0.001	−0.07 (−0.08–0.06)	<0.001	−0.03 (−0.04–0.03)	<0.001
Age	0.98 (0.96–1.00)	0.029	0.87 (0.84–0.91)	<0.001	1.39 (1.34–1.44)	<0.001	1.07 (1.05–1.09)	<0.001	0.05 (0.04–0.05)	<0.001	0.00 (0.00–0.01)	0.403
Elixhauser comorbidity score	2.14 (2.08–2.20)	<0.001	2.91 (2.75–3.09)	<0.001	4.14 (3.97–4.31)	<0.001	2.67 (2.60–2.74)	<0.001	0.39 (0.38–0.39)	<0.001	0.27 (0.26–0.28)	<0.001
COVID-19-related SARI	2.59 (2.34–2.87)	<0.001	8.07 (7.07–9.22)	<0.001	4.44 (3.93–5.00)	<0.001	3.23 (2.94–3.56)	<0.001	0.81 (0.78–0.85)	<0.001	0.51 (0.48–0.54)	<0.001

## Data Availability

The original contributions presented in the study are included in the article/Supplementary Material, further inquiries can be directed to the corresponding author.

## Supplementary Materials

The following supporting information can be downloaded at: https://www.mdpi.com/article/10.3390/vaccines12060634/s1, Supplementary Table S1: ICD-10-based definitions for IC-cohorts. Supplementary Table S2: Proportions of comorbidities in Non-IC patients with COVID-19-related SARI, IC patients with COVID-19-related SARI and IC sub-cohorts with COVID-19-related SARI. Supplementary Table S3: Proportions of comorbidities in IC patients, IC patients with COVID-19-related SARI and IC sub-cohorts with COVID-19-related SARI. Supplementary Table S4: In-hospital outcome for IC-sub-cohorts with and without COVID-19-related SARI.

## Abbreviations

CI	confidence intervals
COVID-19	coronavirus disease 2019
GLMM	generalized linear mixed models
IC	immunocompromised
ICD-10	International Statistical Classification of Diseases and Related Health Problems
ICU	intensive care unit
LMM	linear mixed models
Non IC	non-immunocompromised
OPS	operations and procedures (German adaption)
OR	odds ratio
SARI	severe acute respiratory infection
SARS-CoV-2	severe acute respiratory syndrome coronavirus type 2

## References

[1] Evans R.A., Dube S., Lu Y., Yates M., Arnetorp S., Barnes E., Bell S., Carty L., Evans K., Graham S. (2023). Impact of COVID-19 on immunocompromised populations during the Omicron era: Insights from the observational population-based INFORM study. Lancet Reg. Health Eur..

[2] Flahault A., Calmy A., Costagliola D., Drapkina O., Eckerle I., Larson H.J., Legido-Quigley H., Noakes C., Kazatchkine M., Kluge H. (2023). No time for complacency on COVID-19 in Europe. Lancet.

[3] Jank M., Oechsle A.-L., Armann J., Behrends U., Berner R., Chao C.-M., Diffloth N., Doenhardt M., Hansen G., Hufnagel M. (2023). Comparing SARS-CoV-2 variants among children and adolescents in Germany: Relative risk of COVID-19-related hospitalization, ICU admission and mortality. Infection.

[4] Bonsignore M., Hohenstein S., Kodde C., Leiner J., Schwegmann K., Bollmann A., Möller R., Kuhlen R., Nachtigall I. (2022). Burden of hospital-acquired SARS-CoV-2 infections in Germany: Occurrence and outcomes of different variants. J. Hosp. Infect..

[5] Sakuramoto K., Wada D., Maruyama S., Muroya T., Saito F., Nakamori Y., Kuwagata Y. (2024). Evaluation of characteristics and prognosis of COVID-19 patients requiring invasive mechanical ventilation during dominance of nonvariant, alpha, delta, and omicron variants in tertiary hospitals of Japan. BMC Infect. Dis..

[6] Robert Koch Institut Digitales Impfquoten-Monitoring COVID-19. http://www.rki.de/DE/Content/InfAZ/N/Neuartiges_Coronavirus/Daten/Impfquotenmonitoring.xlsx?__blob=publicationFile.

[7] Bahremand T., Yao J.A., Mill C., Piszczek J., Grant J.M., Smolina K. (2023). COVID-19 hospitalisations in immunocompromised individuals in the Omicron era: A population-based observational study using surveillance data in British Columbia, Canada. Lancet Reg. Health Am..

[8] DeWolf S., Laracy J.C., Perales M.-A., Kamboj M., van den Brink M.R.M., Vardhana S. (2022). SARS-CoV-2 in immunocompromised individuals. Immunity.

[9] Shoham S., Batista C., Ben Amor Y., Ergonul O., Hassanain M., Hotez P., Kang G., Kim J.H., Lall B., Larson H.J. (2023). Vaccines and therapeutics for immunocompromised patients with COVID-19. EClinicalMedicine.

[10] Ketkar A., Willey V., Pollack M., Glasser L., Dobie C., Wenziger C., Teng C.-C., Dube C., Cunningham D., Verduzco-Gutierrez M. (2023). Assessing the risk and costs of COVID-19 in immunocompromised populations in a large United States commercial insurance health plan: The EPOCH-US Study. Curr. Med. Res. Opin..

[11] Lübbert C., Dykukha I., Pelz J.-P., Yearley H., Junker W., Gruber N., Escher S., Biereth K., Melnik S., Puschmann J. (2023). Individuals at risk for severe COVID-19 in whom ritonavir-containing therapies are contraindicated or may lead to interactions with concomitant medications: A retrospective analysis of German health insurance claims data. Drugs Context.

[12] Obeid M., Suffiotti M., Pellaton C., Bouchaab H., Cairoli A., Salvadé V., Stevenel C., Hottinger R., Pythoud C., Coutechier L. (2022). Humoral Responses Against Variants of Concern by COVID-19 mRNA Vaccines in Immunocompromised Patients. JAMA Oncol..

[13] Benning L., Morath C., Bartenschlager M., Kim H., Reineke M., Beimler J., Buylaert M., Nusshag C., Kälble F., Reichel P. (2022). Neutralizing antibody response against the B.1.617.2 (delta) and the B.1.1.529 (omicron) variants after a third mRNA SARS-CoV-2 vaccine dose in kidney transplant recipients. Am. J. Transplant.

[14] Tartof S.Y., Slezak J.M., Puzniak L., Hong V., Frankland T.B., Ackerson B.K., Takhar H.S., Ogun O.A., Simmons S.R., Zamparo J.M. (2022). Effectiveness of a third dose of BNT162b2 mRNA COVID-19 vaccine in a large US health system: A retrospective cohort study. Lancet Reg. Health Am..

[15] Mozaffari E., Chandak A., Gottlieb R.L., Chima-Melton C., Read S.H., Jiang H., Chiang M., Lee E., Gupta R., Berry M. (2023). Remdesivir Reduced Mortality in Immunocompromised Patients Hospitalized for COVID-19 Across Variant Waves: Findings from Routine Clinical Practice. Clin. Infect. Dis..

[16] Antinori A., Bausch-Jurken M. (2023). The Burden of COVID-19 in the Immunocompromised Patient: Implications for Vaccination and Needs for the Future. J. Infect. Dis..

[17] Robert-Koch-Institut Epidemiologisches Bulletin: Aktuelle Daten und Informationen zu Infektionserkrankungen und Public Health. https://www.openagrar.de/receive/zimport_mods_00003614.

[18] Häckl D., Pignot M., Dang P.L., Lauenroth V., Jah F., Wendtner C.-M. (2024). Klinische Verläufe und Kosten für Hospitalisierungen von COVID-19-Patienten mit potenziell eingeschränktem Immunsystem in Deutschland. Dtsch. Med. Wochenschr..

[19] Federal Institute for Drugs and Medical Devices ICD-10-GM: International Statistical Classification of Diseases, German Modification. https://www.bfarm.de/EN/Code-systems/Classifications/ICD/ICD-10-GM/_node.html.

[20] Moore B.J., White S., Washington R., Coenen N., Elixhauser A. (2017). Identifying Increased Risk of Readmission and In-hospital Mortality Using Hospital Administrative Data: The AHRQ Elixhauser Comorbidity Index. Med. Care.

[21] Gasparini A. (2018). Comorbidity: An R package for computing comorbidity scores. JOSS.

[22] Buda S., Tolksdorf K., Schuler E., Kuhlen R., Haas W. (2017). Establishing an ICD-10 code based SARI-surveillance in Germany—Description of the system and first results from five recent influenza seasons. BMC Public Health.

[23] Leiner J., Hohenstein S., Pellissier V., König S., Winklmair C., Nachtigall I., Bollmann A., Kuhlen R. (2023). COVID-19 and Severe Acute Respiratory Infections: Monitoring Trends in 421 German Hospitals During the First Four Pandemic Waves. Infect. Drug Resist..

[24] UK Department of Health & Social Care Defining the Highest-Risk Clinical Subgroups upon Community Infection with SARS-CoV-2 When Considering the Use of Neutralising Monoclonal Antibodies (nMABs) and Antiviral Drugs: Independent Advisory Group Report. https://www.gov.uk/government/publications/higher-risk-patients-eligible-for-covid-19-treatments-independent-advisory-group-report/defining-the-highest-risk-clinical-subgroups-upon-community-infection-with-sars-cov-2-when-considering-the-use-of-neutralising-monoclonal-antibodies.

[25] Federal Institute for Drugs and Medical Devices German Adaptation of the International Classification of the Procedures in Medicine of the World Health Organization, Version. https://www.bfarm.de/SharedDocs/Downloads/DE/Kodiersysteme/klassifikationen/ops/vorgaenger/ops2022_zip.html?nn=841246&cms_dlConfirm=true&cms_calledFromDoc=841246.

[26] WHO Working Group on the Clinical Characterisation and Management of COVID-19 infection (2020). A minimal common outcome measure set for COVID-19 clinical research. Lancet Infect. Dis..

[27] Von Elm E., Altman D.G., Egger M., Pocock S.J., Gøtzsche P.C., Vandenbroucke J.P. (2007). The Strengthening the Reporting of Observational Studies in Epidemiology (STROBE) statement: Guidelines for reporting observational studies. Lancet.

[28] Bates D., Mächler M., Bolker B., Walker S. (2015). Fitting Linear Mixed-Effects Models Using lme4. J. Stat. Soft..

[29] Ketkar A., Willey V., Glasser L., Dobie C., Wenziger C., Teng C.-C., Dube C., Hirpara S., Cunningham D., Verduzco-Gutierrez M. (2024). Assessing the Burden and Cost of COVID-19 across Variants in Commercially Insured Immunocompromised Populations in the United States: Updated Results and Trends from the Ongoing EPOCH-US Study. Adv. Ther..

[30] Nevejan L., Ombelet S., Laenen L., Keyaerts E., Demuyser T., Seyler L., Soetens O., van Nedervelde E., Naesens R., Geysels D. (2022). Severity of COVID-19 among Hospitalized Patients: Omicron Remains a Severe Threat for Immunocompromised Hosts. Viruses.

[31] Singson J.R.C., Kirley P.D., Pham H., Rothrock G., Armistead I., Meek J., Anderson E.J., Reeg L., Lynfield R., Ropp S. (2022). Factors Associated with Severe Outcomes Among Immunocompromised Adults Hospitalized for COVID-19-COVID-NET, 10 States, March 2020-February 2022. MMWR Morb. Mortal. Wkly. Rep..

[32] Khoury E., Nevitt S., Madsen W.R., Turtle L., Davies G., Palmieri C. (2022). Differences in Outcomes and Factors Associated with Mortality Among Patients with SARS-CoV-2 Infection and Cancer Compared with Those Without Cancer: A Systematic Review and Meta-analysis. JAMA Netw. Open.

[33] Vujčić I. (2023). Outcomes of COVID-19 among patients with liver disease. World J. Gastroenterol..

[34] Aleshina O.A., Zakurdaeva K., Vasileva A.N., Dubov S.K., Dubov V.S., Vorobyev V.I., Butaev L.S., Sukhareva A.M., Gavrilova L.V., Toropova I.Y. (2023). Clinical Outcomes in Patients With COVID-19 and Hematologic Disease. Clin. Lymphoma Myeloma Leuk..

[35] Yin Y., Li Y., Liu Y., Fan C., Jiang Y. (2024). Baseline immune status and the effectiveness of response to enteral nutrition among ICU patients with COVID-19: An observational, retrospective study. Nutrition.

[36] Turtle L., Thorpe M., Drake T.M., Swets M., Palmieri C., Russell C.D., Ho A., Aston S., Wootton D.G., Richter A. (2023). Outcome of COVID-19 in hospitalised immunocompromised patients: An analysis of the WHO ISARIC CCP-UK prospective cohort study. PLoS Med..

[37] Bonsignore M., Hohenstein S., Kodde C., Leiner J., Schwegmann K., Bollmann A., Möller R., Kuhlen R., Nachtigall I. (2022). The Disease Course of Hospitalized COVID-19 Patients During the Delta and Omicron Periods in Consideration of Vaccination Status. Dtsch. Arztebl. Int..

[38] Meyerowitz E.A., Scott J., Richterman A., Male V., Cevik M. (2023). Clinical course and management of COVID-19 in the era of widespread population immunity. Nat. Rev. Microbiol..

[39] Embi P.J., Levy M.E., Naleway A.L., Patel P., Gaglani M., Natarajan K., Dascomb K., Ong T.C., Klein N.P., Liao I.-C. (2021). Effectiveness of 2-Dose Vaccination with mRNA COVID-19 Vaccines Against COVID-19-Associated Hospitalizations Among Immunocompromised Adults—Nine States, January-September 2021. MMWR Morb. Mortal. Wkly. Rep..

[40] Soeroto A.Y., Yanto T.A., Kurniawan A., Hariyanto T.I. (2023). Efficacy and safety of tixagevimab-cilgavimab as pre-exposure prophylaxis for COVID-19: A systematic review and meta-analysis. Rev. Med. Virol..

[41] Li H., Ma Q., Ren J., Guo W., Feng K., Li Z., Huang T., Cai Y.-D. (2023). Immune responses of different COVID-19 vaccination strategies by analyzing single-cell RNA sequencing data from multiple tissues using machine learning methods. Front. Genet..

[42] Ren J.-X., Gao Q., Zhou X.-C., Chen L., Guo W., Feng K.-Y., Lu L., Huang T., Cai Y.-D. (2023). Identification of Gene Markers Associated with COVID-19 Severity and Recovery in Different Immune Cell Subtypes. Biology.

[43] Haidar G., Hodges J.C., Bilderback A., Lukanski A., Linstrum K., Postol B., Troyan R., Wisniewski M.K., Coughenour L., Heaps A. (2024). Prospective Assessment of Humoral and Cellular Immune Responses to a Third COVID-19 mRNA Vaccine Dose Among Immunocompromised Individuals. J. Infect. Dis..

[44] Wang X., Haeussler K., Spellman A., Phillips L.E., Ramiller A., Bausch-Jurken M.T., Sharma P., Krivelyova A., Vats S., van de Velde N. (2023). Comparative effectiveness of mRNA-1273 and BNT162b2 COVID-19 vaccines in immunocompromised individuals: A systematic review and meta-analysis using the GRADE framework. Front. Immunol..

[45] Kluge S. (2024). S3-Leitlinie—Empfehlungen zur Therapie von Patienten mit COVID-19. https://register.awmf.org/assets/guidelines/113-001l_S3_Empfehlungen-zur-Therapie-von-Patienten-mit-COVID-19_2024-01.pdf.

[46] Robert-Koch-Institut Deutscher Elektronischer Sequenzdaten-Hub (DESH). https://www.rki.de/DE/Content/InfAZ/N/Neuartiges_Coronavirus/DESH/Schaubild-Phasen-1-und-2.pdf?__blob=publicationFile.

