# Supplementary material for: Retrospective, Observational Analysis on the Impact of SARS-CoV-2 Variant Omicron in Hospitalized Immunocompromised Patients in a German Hospital Network—The VISAGE Study
Source: Vaccines (Basel). 2024 Jun 7;12(6):634. doi: 10.3390/vaccines12060634 (PMC11209028; doi:10.3390/vaccines12060634)
Supplement: Supplementary file 1 [file vaccines-12-00634-s001.zip › vaccines-3002714-supplementary.pdf]

Supplementary **Table S1.** ICD-10-based definitions for IC-cohorts

Disease	Definition
Solid tumors	C00, C01, C02, C03, C04, C05, C06, C07, C08, C09, C10, C11, C12, C13, C14, C15, C16, C17, C18, C19, C20, C21, C22, C23, C24, C25, C26, C30, C31, C32, C33, C34, C37, C38, C39, C40, C41, C43, C45, C46, C47, C48, C49, C50, C51, C52, C53, C54, C55, C56, C57, C58, C60, C61, C62, C63, C64, C65, C66, C67, C68, C69, C70, C71, C72, C73, C74, C75, C76, C97, C77, C78, C79, C80
Hematological diseases	C81-C96 and one of: Z94.80, Z94.81, D90, 8-52, 8-53, 8-541-8-547, 8-549
Solid organ transplants	Z94.0-Z94.4, Z94.88
Patients on hemodialysis	N18.4, N18.5 and Z49
Chronic liver disease	K74, K70.3, K70.41, K70.42, K71.7, K72.1
HIV	B20-B24
Autoimmune diseases	M05, M06, M30, M31.3, M31.5, M31.7, M32, M33, M34, M35.2, D86, L40.0, G35, L10.0, N00.0-N00.7, N01.0-N01.7, N02.0-N02.7, N03.0-N03.7, N04.0-N04.7, N06.0-N06.7, N07.0-N07.7, K75.4
Congenital immunodeficiency	D80-D90

Supplementary **Table S2.** Proportions of comorbidities in Non-IC patients with COVID-19 related SARI, IC patients with COVID-19 related SARI and IC subcohorts with COVID-19 related SARI

	**Non-IC with COVID-19 related SARI**, N = 14,772	**IC with COVID-19 related SARI, ** N = 2,0371	**p-value**	**Solid tumors with COVID-19 related SARI**, N = 1,000	**p-value**	**Hematological diseases with COVID-19 related SARI**, N = 133[1]	**p-value**	**Solid organ transplants with COVID-19 related SARI**, N = 138	**p-value**	**End-stage renal disease with COVID-19 related SARI**, N = 27	**p-value**	**End-stage chronic liver disease with COVID-19 related SARI**, N = 243	**p-value**
Congestive heart failure	4,569 (31%)	586 (29%)	0.050	275 (28%)	0.025	26 (20%)	0.006	41 (30%)	0.829	17 (63%)	< 0.001	104 (43%)	< 0.001
Cardiac arrhythmias	4,970 (34%)	570 (28%)	< 0.001	283 (28%)	< 0.001	31 (23%)	0.015	29 (21%)	0.002	10 (37%)	0.866	79 (33%)	0.762
Valvular disease	1,381 (9.3%)	163 (8.0%)	0.053	80 (8.0%)	0.171	5 (3.8%)	0.039	9 (6.5%)	0.322	4 (15%)	0.520	29 (12%)	0.208
Pulmonary circulation disorders	942 (6.4%)	162 (8.0%)	0.008	85 (8.5%)	0.010	9 (6.8%)	0.996	7 (5.1%)	0.653	4 (15%)	0.162	20 (8.2%)	0.299
Peripheral vascular disorders	1,310 (8.9%)	270 (13%)	< 0.001	154 (15%)	< 0.001	17 (13%)	0.154	10 (7.2%)	0.605	7 (26%)	0.006	46 (19%)	< 0.001
Hypertension, uncomplicated	6,646 (45%)	851 (42%)	0.007	430 (43%)	0.233	46 (35%)	0.021	56 (41%)	0.342	11 (41%)	0.803	83 (34%)	< 0.001
Hypertension, complicated	2,173 (15%)	312 (15%)	0.490	142 (14%)	0.693	14 (11%)	0.217	32 (23%)	0.008	10 (37%)	0.003	47 (19%)	0.054
Paralysis	854 (5.8%)	121 (5.9%)	0.813	57 (5.7%)	0.971	4 (3.0%)	0.238	9 (6.5%)	0.851	0 (0%)	0.382	9 (3.7%)	0.215
Other neurological disorders	1,535 (10%)	232 (11%)	0.181	77 (7.7%)	0.008	3 (2.3%)	0.003	10 (7.2%)	0.286	0 (0%)	0.146	18 (7.4%)	0.159
Chronic pulmonary disease	2,155 (15%)	371 (18%)	< 0.001	204 (20%)	< 0.001	11 (8.3%)	0.053	15 (11%)	0.266	3 (11%)	0.811	37 (15%)	0.851
Diabetes, uncomplicated	2,485 (17%)	350 (17%)	0.708	193 (19%)	0.048	16 (12%)	0.175	19 (14%)	0.400	5 (19%)	1.000	52 (21%)	0.072
Diabetes, complicated	1,806 (12%)	244 (12%)	0.777	104 (10%)	0.096	18 (14%)	0.745	32 (23%)	< 0.001	8 (30%)	0.014	48 (20%)	< 0.001
Hypothyroidism	1,993 (13%)	288 (14%)	0.445	130 (13%)	0.694	18 (14%)	1.000	19 (14%)	1.000	6 (22%)	0.296	43 (18%)	0.071
Renal failure	5,119 (35%)	792 (39%)	< 0.001	321 (32%)	0.107	53 (40%)	0.245	121 (88%)	< 0.001	27 (100%)	< 0.001	107 (44%)	0.003
Liver disease	374 (2.5%)	318 (16%)	< 0.001	69 (6.9%)	< 0.001	10 (7.5%)	< 0.001	17 (12%)	< 0.001	1 (3.7%)	1.000	243 (100%)	< 0.001
Solid tumour without metastasis	0 (0%)	949 (47%)	< 0.001	949 (95%)	< 0.001	5 (3.8%)	< 0.001	4 (2.9%)	< 0.001	1 (3.7%)	< 0.001	33 (14%)	< 0.001
Rheumatoid artritis/collaged vascular disease	80 (0.5%)	334 (16%)	< 0.001	21 (2.1%)	< 0.001	1 (0.8%)	1.000	2 (1.4%)	0.391	1 (3.7%)	0.358	7 (2.9%)	< 0.001
Coagulopathy	656 (4.4%)	236 (12%)	< 0.001	81 (8.1%)	< 0.001	34 (26%)	< 0.001	15 (11%)	< 0.001	3 (11%)	0.226	67 (28%)	< 0.001
Obesity	1,250 (8.5%)	160 (7.9%)	0.377	71 (7.1%)	0.148	11 (8.3%)	1.000	6 (4.3%)	0.115	4 (15%)	0.402	30 (12%)	0.042
Weight loss	1,353 (9.2%)	407 (20%)	< 0.001	254 (25%)	< 0.001	30 (23%)	< 0.001	12 (8.7%)	0.968	3 (11%)	0.986	73 (30%)	< 0.001
Fluid and electrolyte disorders	7,698 (52%)	1,092 (54%)	0.214	548 (55%)	0.106	62 (47%)	0.240	61 (44%)	0.077	10 (37%)	0.170	163 (67%)	< 0.001

Supplementary **Table S2-continued.** Proportions of comorbidities in Non-IC patients with COVID-19 related SARI, IC patients with COVID-19 related SARI and IC subcohorts with COVID-19 related SARI

**HIV with COVID-19 related SARI**, N = 7	**p-value**	**Automimmune disease with COVID-19 related SARI**, N = 494	**p-value**	**Congenital immunodefiency with COVID-19 related SARI**, N = 347	**p-value**
0 (0%)	0.173	134 (27%)	0.080	87 (25%)	0.023
0 (0%)	0.138	138 (28%)	0.009	92 (27%)	0.006
0 (0%)	0.841	39 (7.9%)	0.310	21 (6.1%)	0.046
0 (0%)	1.000	37 (7.5%)	0.368	29 (8.4%)	0.169
1 (14%)	1.000	39 (7.9%)	0.503	38 (11%)	0.211
0 (0%)	0.044	226 (46%)	0.774	150 (43%)	0.550
0 (0%)	0.572	70 (14%)	0.788	53 (15%)	0.829
1 (14%)	0.878	43 (8.7%)	0.009	13 (3.7%)	0.135
1 (14%)	1.000	123 (25%)	< 0.001	25 (7.2%)	0.066
2 (29%)	0.608	88 (18%)	0.054	64 (18%)	0.054
0 (0%)	0.494	71 (14%)	0.170	57 (16%)	0.903
0 (0%)	0.682	44 (8.9%)	0.031	34 (9.8%)	0.199
1 (14%)	1.000	75 (15%)	0.311	58 (17%)	0.098
0 (0%)	0.126	173 (35%)	0.904	132 (38%)	0.210
3 (43%)	< 0.001	27 (5.5%)	< 0.001	19 (5.5%)	0.001
0 (0%)		17 (3.4%)	< 0.001	49 (14%)	< 0.001
0 (0%)	1.000	325 (66%)	< 0.001	61 (18%)	< 0.001
2 (29%)	0.029	37 (7.5%)	0.002	41 (12%)	< 0.001
1 (14%)	1.000	40 (8.1%)	0.838	29 (8.4%)	1.000
1 (14%)	1.000	51 (10%)	0.423	54 (16%)	< 0.001
5 (71%)	0.519	247 (50%)	0.380	168 (48%)	0.191

Supplementary **Table S3.** Proportions of comorbidities in IC patients, IC patients with COVID-19 related SARI and IC subcohorts with COVID-19 related SARI

	**IC**, N = 129,515	**IC with COVID-19 related SARI** , N = 2,037	**p-value**	**Solid tumors**, N = 92,376	**Solid tumors with COVID-19 related SARI**, N = 1,000	**p-value**	**Hemato-logical diseases**, N = 5,217	**Hemato-logical diseases with COVID-19 related SARI**, N = 133	**p-value**	Solid organ transplants, N = 1,842	**Solid organ transplants with COVID-19 related SARI**, N = 138	**p-value**
Congestive heart failure	15,436 (12%)	586 (29%)	< 0.001	9,384 (10%)	275 (28%)	< 0.001	450 (8.6%)	26 (20%)	< 0.001	326 (18%)	41 (30%)	< 0.001
Cardiac arrhythmias	19,664 (15%)	570 (28%)	< 0.001	13,382 (14%)	283 (28%)	< 0.001	523 (10%)	31 (23%)	< 0.001	342 (19%)	29 (21%)	0.550
Valvular disease	6,276 (4.8%)	163 (8.0%)	< 0.001	3,774 (4.1%)	80 (8.0%)	< 0.001	217 (4.2%)	5 (3.8%)	0.993	154 (8.4%)	9 (6.5%)	0.550
Pulmonary circulation disorders	2,857 (2.2%)	162 (8.0%)	< 0.001	1,757 (1.9%)	85 (8.5%)	< 0.001	56 (1.1%)	9 (6.8%)	< 0.001	77 (4.2%)	7 (5.1%)	0.777
Peripheral vascular disorders	18,969 (15%)	270 (13%)	0.083	15,446 (17%)	154 (15%)	0.284	772 (15%)	17 (13%)	0.601	259 (14%)	10 (7.2%)	0.034
Hypertension, uncomplicated	56,468 (44%)	851 (42%)	0.104	41,439 (45%)	430 (43%)	0.253	1,664 (32%)	46 (35%)	0.573	763 (41%)	56 (41%)	0.917
Hypertension, complicated	10,541 (8.1%)	312 (15%)	< 0.001	6,524 (7.1%)	142 (14%)	< 0.001	185 (3.5%)	14 (11%)	< 0.001	322 (17%)	32 (23%)	0.116
Other neurological disorders	8,903 (6.9%)	232 (11%)	< 0.001	3,724 (4.0%)	77 (7.7%)	< 0.001	156 (3.0%)	3 (2.3%)	0.815	76 (4.1%)	10 (7.2%)	0.129
Chronic pulmonary disease	14,382 (11%)	371 (18%)	< 0.001	10,294 (11%)	204 (20%)	< 0.001	269 (5.2%)	11 (8.3%)	0.163	116 (6.3%)	15 (11%)	0.057
Diabetes, uncomplicated	19,033 (15%)	350 (17%)	0.002	13,663 (15%)	193 (19%)	< 0.001	568 (11%)	16 (12%)	0.782	260 (14%)	19 (14%)	1.000
Diabetes, complicated	8,262 (6.4%)	244 (12%)	< 0.001	4,990 (5.4%)	104 (10%)	< 0.001	257 (4.9%)	18 (14%)	< 0.001	251 (14%)	32 (23%)	0.003
Hypothyroidism	15,832 (12%)	288 (14%)	0.010	10,913 (12%)	130 (13%)	0.269	417 (8.0%)	18 (14%)	0.032	235 (13%)	19 (14%)	0.833
Renal failure	29,061 (22%)	792 (39%)	< 0.001	19,051 (21%)	321 (32%)	< 0.001	1,269 (24%)	53 (40%)	< 0.001	1,245 (68%)	121 (88%)	< 0.001
Liver disease	14,354 (11%)	318 (16%)	< 0.001	3,954 (4.3%)	69 (6.9%)	< 0.001	99 (1.9%)	10 (7.5%)	< 0.001	324 (18%)	17 (12%)	0.143
Lymphoma	4,114 (3.2%)	102 (5.0%)	< 0.001	860 (0.9%)	27 (2.7%)	< 0.001	3,409 (65%)	71 (53%)	0.006	44 (2.4%)	0 (0%)	0.124
Metastatic cancer	38,373 (30%)	431 (21%)	< 0.001	38,373 (42%)	431 (43%)	0.335	264 (5.1%)	8 (6.0%)	0.768	57 (3.1%)	0 (0%)	0.067
Solid tumour without metastasis	89,690 (69%)	949 (47%)	< 0.001	89,690 (97%)	949 (95%)	< 0.001	233 (4.5%)	5 (3.8%)	0.859	168 (9.1%)	4 (2.9%)	0.019
Rheumatoid artritis/collaged vascular disease	13,493 (10%)	334 (16%)	< 0.001	1,057 (1.1%)	21 (2.1%)	0.008	173 (3.3%)	1 (0.8%)	0.162	25 (1.4%)	2 (1.4%)	1.000
Coagulopathy	5,500 (4.2%)	236 (12%)	< 0.001	2,685 (2.9%)	81 (8.1%)	< 0.001	696 (13%)	34 (26%)	< 0.001	81 (4.4%)	15 (11%)	0.001
Obesity	12,526 (9.7%)	160 (7.9%)	0.007	8,070 (8.7%)	71 (7.1%)	0.077	362 (6.9%)	11 (8.3%)	0.672	133 (7.2%)	6 (4.3%)	0.271
Weight loss	15,223 (12%)	407 (20%)	< 0.001	12,000 (13%)	254 (25%)	< 0.001	687 (13%)	30 (23%)	0.003	115 (6.2%)	12 (8.7%)	0.340
Fluid and electrolyte disorders	22,950 (18%)	1,092 (54%)	< 0.001	15,007 (16%)	548 (55%)	< 0.001	862 (17%)	62 (47%)	< 0.001	408 (22%)	61 (44%)	< 0.001

Supplementary **Table S3 - continued.** Proportions of comorbidities in IC patients, IC patients with COVID-19 related SARI and IC subcohorts with COVID-19 related SARI

Patients on hemodialysis, N = 709	Patients on hemodialysis with COVID-19 related SARI, N = 27	p-value	End-stage Chronic liver diseases, N = 11,040	End-stage Chronic liver diseases with COVID-19 related SARI , N = 243	p-value	HIV, N = 176	HIV with COVID-19 related SARI, N = 7	p-value	Autoimmune diseases, N = 20,976	Autoimmune diseases with COVID-19 related SARI, N = 494	p-value	Congenital immuno-deficiency, N = 6,508	Congenital immuno-deficiency with COVID-19 related SARI, N = 347	p-value
272 (38%)	17 (63%)	0.018	2,475 (22%)	104 (43%)	< 0.001	12 (6.8%)	0 (0%)	1.000	3,042 (15%)	134 (27%)	< 0.001	796 (12%)	87 (25%)	< 0.001
229 (32%)	10 (37%)	0.759	2,285 (21%)	79 (33%)	< 0.001	6 (3.4%)	0 (0%)	1.000	3,481 (17%)	138 (28%)	< 0.001	897 (14%)	92 (27%)	< 0.001
108 (15%)	4 (15%)	1.000	907 (8.2%)	29 (12%)	0.050	5 (2.8%)	0 (0%)	1.000	1,277 (6.1%)	39 (7.9%)	0.119	330 (5.1%)	21 (6.1%)	0.495
29 (4.1%)	4 (15%)	0.030	412 (3.7%)	20 (8.2%)	< 0.001	2 (1.1%)	0 (0%)	1.000	603 (2.9%)	37 (7.5%)	< 0.001	175 (2.7%)	29 (8.4%)	< 0.001
185 (26%)	7 (26%)	1.000	1,160 (11%)	46 (19%)	< 0.001	8 (4.5%)	1 (14%)	0.781	1,555 (7.4%)	39 (7.9%)	0.751	801 (12%)	38 (11%)	0.505
311 (44%)	11 (41%)	0.902	4,154 (38%)	83 (34%)	0.299	43 (24%)	0 (0%)	0.298	9,420 (45%)	226 (46%)	0.745	2,119 (33%)	150 (43%)	< 0.001
264 (37%)	10 (37%)	1.000	1,519 (14%)	47 (19%)	0.017	7 (4.0%)	0 (0%)	1.000	2,114 (10%)	70 (14%)	0.004	483 (7.4%)	53 (15%)	< 0.001
24 (3.4%)	0 (0%)	0.674	654 (5.9%)	18 (7.4%)	0.407	13 (7.4%)	1 (14%)	1.000	4,576 (22%)	123 (25%)	0.113	250 (3.8%)	25 (7.2%)	0.003
84 (12%)	3 (11%)	1.000	1,293 (12%)	37 (15%)	0.114	19 (11%)	2 (29%)	0.400	2,663 (13%)	88 (18%)	< 0.001	716 (11%)	64 (18%)	< 0.001
70 (9.9%)	5 (19%)	0.257	2,447 (22%)	52 (21%)	0.837	11 (6.3%)	0 (0%)	1.000	2,700 (13%)	71 (14%)	0.360	727 (11%)	57 (16%)	0.004
238 (34%)	8 (30%)	0.827	1,544 (14%)	48 (20%)	0.014	6 (3.4%)	0 (0%)	1.000	1,330 (6.3%)	44 (8.9%)	0.027	371 (5.7%)	34 (9.8%)	0.002
109 (15%)	6 (22%)	0.489	1,520 (14%)	43 (18%)	0.097	7 (4.0%)	1 (14%)	0.715	3,019 (14%)	75 (15%)	0.668	654 (10%)	58 (17%)	< 0.001
709 (100%)	27 (100%)		3,452 (31%)	107 (44%)	< 0.001	28 (16%)	0 (0%)	0.541	4,367 (21%)	173 (35%)	< 0.001	1,260 (19%)	132 (38%)	< 0.001
37 (5.2%)	1 (3.7%)	1.000	11,040 (100%)	243 (100%)		18 (10%)	3 (43%)	0.040	1,013 (4.8%)	27 (5.5%)	0.586	302 (4.6%)	19 (5.5%)	0.557
6 (0.8%)	0 (0%)	1.000	73 (0.7%)	7 (2.9%)	< 0.001	11 (6.3%)	1 (14%)	0.949	150 (0.7%)	3 (0.6%)	0.991	505 (7.8%)	38 (11%)	0.041
11 (1.6%)	0 (0%)	1.000	638 (5.8%)	17 (7.0%)	0.507	4 (2.3%)	0 (0%)	1.000	503 (2.4%)	9 (1.8%)	0.496	667 (10%)	28 (8.1%)	0.223
41 (5.8%)	1 (3.7%)	0.973	1,592 (14%)	33 (14%)	0.782	15 (8.5%)	0 (0%)	0.917	1,284 (6.1%)	17 (3.4%)	0.018	1,418 (22%)	49 (14%)	< 0.001
19 (2.7%)	1 (3.7%)	1.000	183 (1.7%)	7 (2.9%)	0.225	0 (0%)	0 (0%)		12,933 (62%)	325 (66%)	0.069	959 (15%)	61 (18%)	0.170
32 (4.5%)	3 (11%)	0.263	1,716 (16%)	67 (28%)	< 0.001	7 (4.0%)	2 (29%)	0.039	591 (2.8%)	37 (7.5%)	< 0.001	296 (4.5%)	41 (12%)	< 0.001
109 (15%)	4 (15%)	1.000	1,313 (12%)	30 (12%)	0.908	9 (5.1%)	1 (14%)	0.842	2,813 (13%)	40 (8.1%)	< 0.001	629 (9.7%)	29 (8.4%)	0.476
48 (6.8%)	3 (11%)	0.627	1,888 (17%)	73 (30%)	< 0.001	18 (10%)	1 (14%)	1.000	1,100 (5.2%)	51 (10%)	< 0.001	599 (9.2%)	54 (16%)	< 0.001
217 (31%)	10 (37%)	0.619	4,062 (37%)	163 (67%)	< 0.001	36 (20%)	5 (71%)	0.007	3,082 (15%)	247 (50%)	< 0.001	1,077 (17%)	168 (48%)	< 0.001

Supplementary **Table S4.** In-hospital outcome for IC-subcohorts with and without COVID-19 related SARI

	Solid tumors, N = 92,376[1]	**Solid tumors with COVID-19 related SARI**, N = 1,000[1]	**Odds ratio [95% CI]**	**p-value**	Hematological diseases, N = 5,217[1]	**Hematological diseases with COVID-19 related SARI**, N = 133[1]	**Odds ratio [95% CI]**	**p-value**	Solid organ transplant, N = 1,842[1]	**Solid organ transplants with COVID-19 related SARI**, N = 138[1]	**Odds ratio [95% CI]**	**p-value**	Patients on hemodialysis, N = 709[1]	**Patients on hemodialysis with COVID-19 related SARI**, N = 27[1]	**Odds ratio [95% CI]**	**p-value**
Intensive care (n(%))	9,429 (10%)	249 (25%)	3.1 [2.3-3.1]	< 0.001	139 (2.7%)	35 (26%)	11 [6.9-17]	< 0.001	244 (13%)	44 (32%)	2.8 [1.8-4.2]	< 0.001	95 (13%)	9 (33%)	3.8 [1.5-9.4]	0.004
Mechanical ventilation (n(%))	1,426 (1.5%)	145 (15%)	10.0 [8.4-12]	< 0.001	43 (0.8%)	24 (18%)	26 [15-45]	< 0.001	34 (1.8%)	26 (19%)	12 [7.2-21]	< 0.001	29 (4.1%)	7 (26%)	11 [3.6-31]	< 0.001
Severe course (n(%))	13,448 (15%)	439 (45%)	4.0 [3.7-4.9]	< 0.001	207 (4.0%)	50 (38%)	12 [8.0-18]	< 0.001	270 (15%)	47 (37%)	2.9 [2.9-4.4]	< 0.001	109 (16%)	14 (52%)	7.2 [3.0-17]	< 0.001
N/A	2,278	27			41	1			69	10			13	0		
In-hospital mortality (n(%))	4,696 (5.3%)	275 (29%)	6.0 [6.0-8.0]	< 0.001	107 (2.1%)	33 (25%)	15 [9.3-24]	< 0.001	42 (2.4%)	17 (15%)	6.8 [3.7-13]	< 0.001	32 (4.6%)	6 (24%)	7.0 [2.5-20]	< 0.001
N/A	2,983	52			54	3			110	21			19	2		
Length of stay (d)	5.9 (7.1)	17.0 (16.0)			6.9 (9.3)	19.8 (17.1)			5.5 (8.1)	11.8 (12.2)			6.8 (9.6)	18.3 (17.1)		
Costs (€)	5,718.3 (6,137.7)	12,611.9 (19,733.9)			8,688.6 (18,193.4)	17,857.8 (20,548.9)			5,384.0 (7,442.9)	11,232.6 (13,757.8)			6,902.1 (11,754.7)	16,688.0 (30,440.2)		

Supplementary **Table S4 – continued.** In-hospital outcome for IC-subcohorts with and without COVID-19 related SARI

End-stage chronic liver diseases, N = 11,040	**End-stage chronic liver diseases with COVID-19 related SARI**, N = 243	**Odds ratio [95% CI]**	**p-value**	HIV, N = 176	**HIV with COVID-19 related SARI**, N = 7	**Odds ratio [95% CI]**	**p-value**	Autoimmune diseases, N = 20,976	**Autoimmune diseases with COVID-19 related SARI**, N = 494	**Odds ratio [95% CI]**	**p-value**	Congenital immuno-deficiency, N = 6,508	**Congenital immune-deficiency with COVID-19 related SARI**, N = 347	**Odds ratio [95% CI]**	**p-value**
1,935 (18%)	103 (42%)	3.4 [2.6-4.5]	< 0.001	21 (12%)	3 (43%)	5.5 [1.2-26]	0.032	1,823 (8.7%)	132 (27%)	3.1 [2.5-3.9]	< 0.001	511 (7.9%)	94 (27%)	3.8 [2.9-5.0]	< 0.001
468 (4.2%)	53 (22%)	6.4 [4.7-8.9]	< 0.001	4 (2.3%)	2 (29%)	17[2.5-117]	0.004	441 (2.1%)	79 (16%)	9.3 [7.1-12]	< 0.001	167 (2.6%)	59 (17%)	7.0 [5.0-9.9]	< 0.001
2,448 (23%)	137 (57%)	4.5 [3.5-5.9]	< 0.001	22 (13%)	3 (43%)	5.1 [0.95-27]	0.057	2,182 (11%)	164 (34%)	3.6 [2.9-4.3]	< 0.001	784 (12%)	120 (36%)	3.7 [2.9-4.8]	< 0.001
358	4			10	0			483	7			143	9		
946 (9.1%)	76 (33%)	5.2 [3.9-6.9]	< 0.001	2 (1.2%)	1 (14%)	14 [1.1-170]	0.044	323 (1.6%)	71 (15%)	9.5 [7.1-13]	< 0.001	264 (4.2%)	58 (18%)	6.2 [4.4-8.8]	< 0.001
618	16			12	0			655	24			194	20		
7.6 (8.0)	19.5 (18.2)			9.2 (15.7)	19.3 (18.0)			6.1 (6.9)	12.7 (12.9)			5.7 (7.4)	13.3 (12.8)		
5,524.3 (5,932.4)	15,673.2 (20,015.1)			6,540.5 (8,444.6)	19,703.3 (22,122.9)			4,825.4 (5,260.5)	9,835.0 (16,659.7)			5,281.5 (6,802.7)	11,488.5 (18,718.4)		
